# Outcomes of audio-instructed and video-instructed dispatcher-assisted cardiopulmonary resuscitation: a systematic review and meta-analysis

**DOI:** 10.1080/07853890.2022.2032314

**Published:** 2022-02-02

**Authors:** Karol Bielski, Bernd W. Böttiger, Michal Pruc, Aleksandra Gasecka, Mariusz Sieminski, Milosz J. Jaguszewski, Jacek Smereka, Natasza Gilis-Malinowska, Frank W. Peacock, Lukasz Szarpak

**Affiliations:** a Institute of Outcomes Research, Polonia University, Czestochowa, Poland; b Polish Society of Disaster Medicine, Research Unit, Warsaw, Poland; c Department of Anesthesiology and Intensive Care Medicine, Faculty of Medicine and University Hospital of Cologne, University of Cologne, Cologne, Germany; d Laboratory of Experimental Clinical Chemistry, Amsterdam University Medical Center, Amsterdam, the Netherlands; e First Chair and Department of Cardiology, Medical University of Warsaw, Warsaw, Poland; f Department of Emergency Medicine, Medical University of Gdansk, Gdansk, Poland; g First Department of Cardiology, Medical University of Gdansk, Gdansk, Poland; h Department of Emergency Medical Service, Wroclaw Medical University, Wroclaw, Poland; i Henry JN Taub Department of Emergency Medicine, Baylor College of Medicine Houston, Houston, TX, United States; j Institute of Outcomes Research, Maria Sklodowska-Curie Medical Academy, Warsaw, Poland; k Research Unit, Maria Sklodowska-Curie Bialystok Oncology Center, Bialystok, Poland

**Keywords:** Emergency medical dispatcher, video-call, cardiac arrest, cardiopulmonary resuscitation, systematic review, meta-analysis

## Abstract

**Background:**

The present meta-analysis of clinical and simulation trials aimed to compare video-instructed dispatcher-assisted bystander cardiopulmonary resuscitation (V-DACPR) with conventional audio-instructed dispatcher-assisted bystander cardiopulmonary resuscitation (C-DACPR).

**Methods:**

We searched PubMed, Embase, Web of Science, Cochrane Collaboration databases and Scopus from inception until June 10, 2021. The primary outcomes were the prehospital return of spontaneous circulation (ROSC), survival to hospital discharge, and survival to hospital discharge with a good neurological outcome for clinical trials, and chest compression quality for simulation trials. Odds ratios (ORs) and mean differences (MDs) with 95% confidence intervals (CIs) indicated the pooled effect. The analyses were performed with the RevMan 5.4 and STATA 14 software.

**Results:**

Overall, 2 clinical and 8 simulation trials were included in this meta-analysis. In clinical trials, C-DACPR and V-DACPR were characterised by, respectively, 11.8% vs. 24.3% of prehospital ROSC (OR = 0.46; 95% CI: 0.30, 0.69; *I*^2^ = 66%; *p* < .001), 10.7% vs. 22.3% of survival to hospital discharge (OR = 0.46; 95% CI: 0.30, 0.70; *I*^2^ = 69%; *p* < .001), and 6.3% vs. 16.0% of survival to hospital discharge with a good neurological outcome (OR = 0.39; 95% CI: 0.23, 0.67; *I*^2^ = 73%; *p* < .001). In simulation trials, chest compression rate per minute equalled 91.3 ± 22.6 for C-DACPR and 107.8 ± 12.6 for V-DACPR (MD = −13.40; 95% CI: −21.86, −4.95; *I*^2^ = 97%; *p* = .002). The respective values for chest compression depth were 38.7 ± 14.3 and 41.8 ± 12.5 mm (MD = −2.67; 95% CI: −8.35, 3.01; *I*^2^ = 98%; *p* = .36).

**Conclusions:**

As compared with C-DACPR, V-DACPR significantly increased prehospital ROSC and survival to hospital discharge. Under simulated resuscitation conditions, V-DACPR exhibited a higher rate of adequate chest compressions than C-DACPR.

**Key messages**
- Bystander cardiopulmonary resuscitation parameters significantly depend on the dispatcher’s support and the manner of the support provided.
- Video-instructed dispatcher-assisted bystander cardiopulmonary resuscitation can increase the rate of prehospital return of spontaneous circulation and survival to hospital discharge.
- Video-instructed dispatcher-assisted bystander cardiopulmonary resuscitation improves the quality of chest compressions compared with dispatcher-assisted resuscitation without video instruction.

## 1. Introduction

Out-of-hospital cardiac arrest (OHCA) is the third leading cause of death in industrialised nations, resulting in more than 700,000 deaths in Europe and in the United States every year [1]. The outcome of OHCA is generally very poor, with less than 10% long-term survival in most countries and settings [2,3].

So far, the most effective intervention to improve OHCA outcomes is immediate cardiopulmonary resuscitation (CPR) performed by bystanders [4–6]. Lay bystander CPR instantaneously provides blood flow and oxygen to the brain and is thus associated with a 2–3-fold increase in long-term survival [4,5]. It is much more effective than any other intervention following OHCA [4,7]. One important challenge for bystanders is the adequate detection of OHCA [7]. Many lay people fear that they might harm the patient and therefore they simply wait for the arrival of emergency medical service, not realising that the brain is already dying [2,7]. Lay bystander activities can be markedly influenced and improved by national CPR educational programs and school children education in CPR (“KIDS SAVE LIVES” initiatives etc.). They can also be immediately enhanced by the conventional audio-instructed dispatcher-assisted bystander CPR (C-DACPR) (“telephone CPR”) and/or by improved technologies and broader nationwide Internet connections – video-instructed dispatcher-assisted bystander CPR (V-DACPR) [4,7–9]) *via* smartphone technologies.

Lay CPR, school children education in resuscitation, and dispatcher-assisted CPR are parts of the BIG FIVE to markedly improve survival in OHCA and resuscitation [4], and they are strongly recommended in the new European Resuscitation Council guidelines on CPR and post-resuscitation care (Systems Saving Lives) [7].

Despite its high effectiveness combined with low efforts and costs and a number needed to treat around 10 [4], dispatcher-assisted telephone CPR is not very common, even in most developed countries [2]. V-DACPR, which has been available for a few years, maybe even more effective than C-DACPR because the dispatcher can see and hear what is going on at the scene and thus can provide even more precise instructions and feedback to lay rescuers [10].

Several recent studies, including clinical trials, have focussed on these topics and showed impressive results with both audio and video techniques [10–19]. Until today, no meta-analysis has investigated the results of all available clinical and simulation trials. Therefore, we conducted this meta-analysis to compare the effects of C-DACPR and V-DACPR in cardiac arrest in these settings.

## 2. Methods

### 2.1. Search strategy

In this review, we followed the Preferred Reporting Items for Systematic Reviews and Meta-Analyses (PRISMA) statement guidelines [20]. The study protocol was not registered. Five online databases (PubMed, Embase, Web of Science, Cochrane Collaboration databases, and Scopus) were searched for papers comparing C-DACPR and V-DACPR. The search was performed with the following terms: “cardiopulmonary resuscitation” OR “CPR” OR “cardiac arrest” AND “survival” OR “mortality” OR “chest compression” OR “outcome” OR “quality” AND “video-assisted” OR “audio assisted” OR “dispatcher” OR “smartphone assisted” OR “audio instruction” OR “video instruction” OR “cell phone assisted.” Two independent reviewers (KB and MP) searched for observational research published from database inception to June 10, 2021.

### 2.2. Study eligibility criteria

Studies included in this meta-analysis met the following PICOS criteria: (1) PARTICIPANTS: simulation or clinical adult cardiac arrest; (2) INTERVENTION: C-DACPR; (3) COMPARISON: V-DACPR; (4) OUTCOMES: detailed information on mortality (clinical trials) or chest compression quality (simulation trials); (5) STUDY DESIGN: randomised controlled trials and observational studies. The exclusion criteria were as follows: (A) studies including paediatric patients; (B) letter to editor, correspondence, editorial; (C) conference abstract; (D) guidelines. Studies were also excluded if the full paper was not available in English.

### 2.3. Data extraction and quality assessment

Two reviewers (KB and MP) independently searched the databases for eligible trials. The following information and relevant data were extracted: the name of the first author, year of publication, country of research, study type. For clinical trials, patient characteristics including sex, age, comorbidities, initial electrocardiogram, as well as resuscitation outcomes were collected. For simulation trials, the number of participants, sex, age, and chest compression parameters were extracted. If a consensus could not be reached, disagreements were resolved by referral to another author (LS).

The risk of bias of the included studies was independently assessed by 3 reviewers (KB, AG, and MJJ) in accordance with a revised tool for risk of bias evaluation in randomised trials (RoB 2) [21] and a tool for assessing the risk of bias in non-randomised studies of interventions (ROBINS-I) [22]. All disagreements were resolved by referral to another author (J.S.). ROBINS-I examines 7 domains of bias: (1) bias due to confounding; (2) bias due to the selection of participants; (3) bias in the classification of interventions; (4) bias due to deviations from the intended interventions; (5) bias due to missing data; (6) bias in the measurement of outcomes; and (7) bias in the selection of the reported results. The overall ROBINS-I judgement at the domain and study level was attributed in accordance with the criteria specified in the Risk-Of-Bias Visualisation (robvis) tool [23].

### 2.4. Statistical analysis

All statistical analyses were carried out by using the Review Manager software version 5.4 (Nordic Cochrane Centre, Cochrane Collaboration) and the STATA 14 software (StataCorp LP, College Station, TX, USA). The significance level for all statistical tests was set at *p* < .05 (2-tailed). For dichotomous data, we used odds ratios (ORs) as the effect measure with 95% confidence intervals (CIs), and for continuous data, mean differences (MDs) with 95% CI were applied. When the continuous outcome was reported in a study as median, range, and interquartile range, we estimated means and standard deviations using the formula described by Hozo et al. [24]. For the meta-analysis, we used the random-effects model (assuming a distribution of effects across studies) to weight estimates of studies in proportion to their significance [25]. Heterogeneity was assessed with the *I*^2^ statistic, in which the results ranged from 0% to 100%. Heterogeneity was interpreted as not observed when *I*^2^ = 0%, low when *I*^2^ = 25%, medium when *I*^2^ = 50%, and high when *I*^2^ = 75% [26].

## 3. Results

### 3.1. Study characteristics

**Figure 1.**
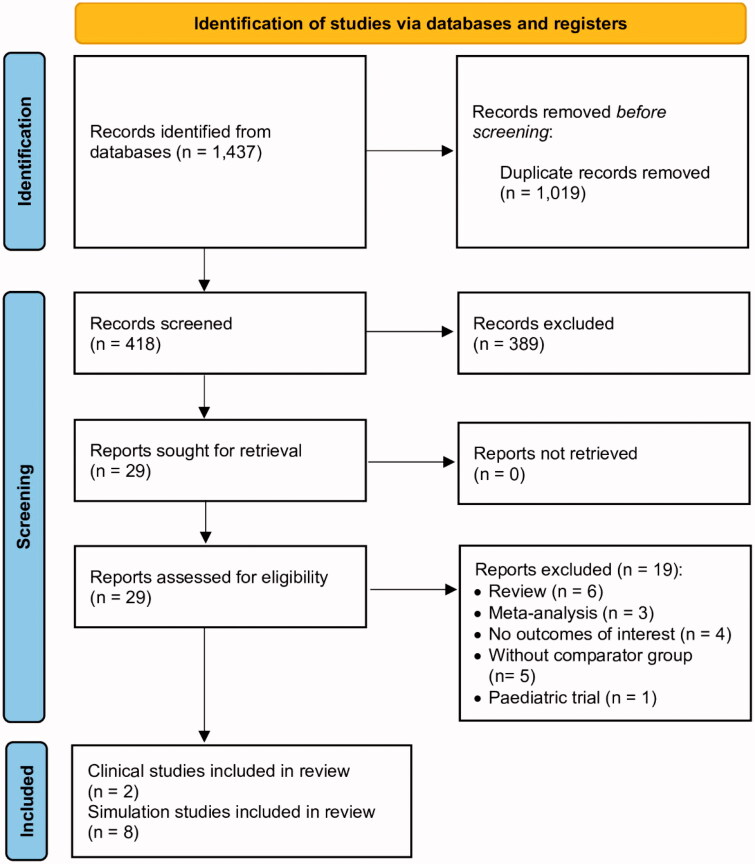
Meta-analysis flow chart of included and excluded studies.

The literature search was carried out in the PubMed, Embase, Web of Science, Cochrane Collaboration databases, and Scopus databases. Initially, 1437 studies were retrieved from database searching. We excluded 1019 duplicate records by checking the author name, publication date, and journal-title. In addition, further 389 records were excluded on the basis of title and abstract. Overall, 29 articles were assessed for eligibility. After the assessment, 19 articles were excluded (4 of them did not report the outcomes of interest). Finally, 10 articles (2 clinical trials [11,12] and 8 simulation trials [10,13–19]) were included in the meta-analysis. Figure 1 presents the study flowchart. The details of the selected trials are summarised in Table 1.

**Table 1.** Characteristics of included studies.

			C-DACPR			V-DACPR		
Study	Country	Study design	No.	Age, years	Male sex, *n* (%)	No.	Age, years	Male sex, *n* (%)
Clinical trials								
Lee et al. 2020 [11]	Korea	A retrospective cohort study	1482	73.5 ± 5.8	925 (62.4)	231	72.5 ± 5.8	156 (67.5)
Lee et al. 2021 [12]	Korea	A retrospective cohort study	1722	72.2 ± 14.7	1087 (63.1)	387	64.9 ± 16.2	263 (68.0)
Simulation trials								
Bolle et al. 2009 [13]	Norway	A randomised controlled simulation study	26	20.95 ± 4.7	9 (34.6)	29	20.9 ± 5.5	8 (27.8)
Choa et al. 2008 [14]	Korea	A single-blind cluster randomised trial	41	32.95 ± 9.5	20 (48.8)	44	31.3 ± 8.4	20 (45.5)
Ecker et al. 2020 [10]	Germany	A randomised controlled simulation study	50	37.6 ± 13.9	15 (30.0)	50	32.92 ± 12.5	19 (38.0)
Hunt et al. 2015 [15]	USA	A prospective randomised controlled study	15	29.0 ± 1.6	5 (33.3)	16	34.4 ± 3.5	4 (25.0)
Lee et al. 2011 [16]	Korea	A randomised controlled simulation study	39	55.3 ± 6.2	19 (48.7)	39	56.6 ± 7.2	20 (51.3)
Lee et al. 2021 [17]	Korea	A randomised controlled simulation study	43	30.8 ± 12.1	9 (20.9)	88	29.8 ± 11.1	21 (23.9)
Stipulante et al. 2016 [18]	Belgium	A prospective randomised study	60	NR	29 (48.3)	60	NR	28 (46.7)
Yang et al. 2009 [19]	Taiwan	A randomised controlled simulation study	53	50.4 ± 12.7	NR	43	50.1 ± 11.5	NR

C-DACPR: conventional audio-instructed dispatcher-assisted bystander cardiopulmonary resuscitation; V-DACPR: video-instructed dispatcher-assisted bystander cardiopulmonary resuscitation; NR: not reported.

### 3.2. Article quality

The Cochrane risk of bias of the included studies is shown in Figures S1 and S2. In the clinical trials, the overall risk of bias was judged as low in one study [11], and reviewers indicated some concerns about the other one [12]. In the case of the simulation trials, 7 studies were categorised as having a low overall risk of bias and 1 was attributed some concerns.

**Figure 2.**
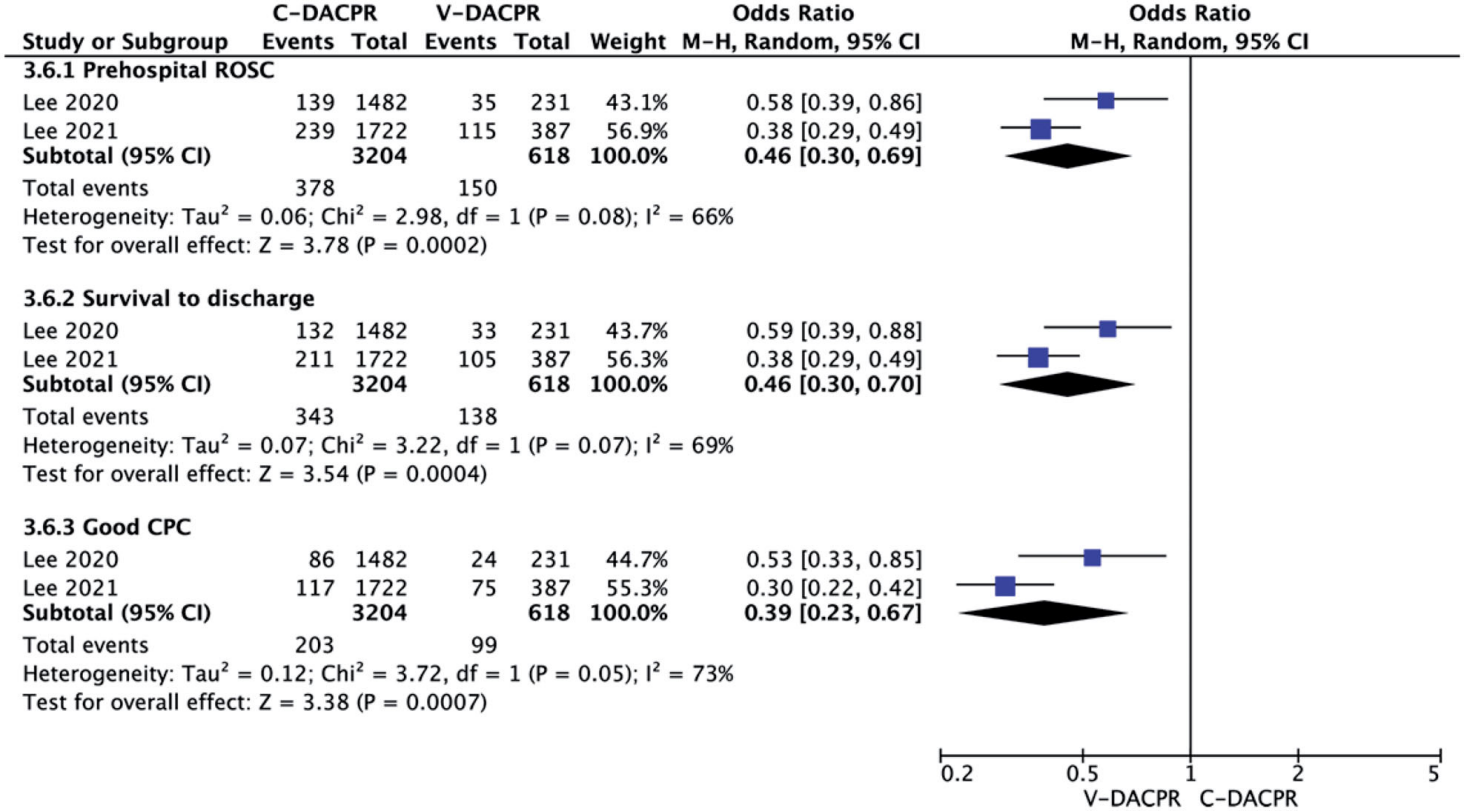
Forest plot of resuscitation outcomes: prehospital ROSC, survival to discharge, good CPC outcome. The centre of each square represents the weighted odds ratios for individual trials, and the corresponding horizontal line stands for 95% CI. The diamonds represent pooled results. C-DACPR: conventional audio-instructed dispatcher-assisted bystander cardiopulmonary resuscitation; V-DACPR: video-instructed dispatcher-assisted bystander cardiopulmonary resuscitation; M–H:? ??; CI: confidence interval; ROSC: return of spontaneous circulation; CPC: Cerebral Performance Categories Scale.

### 3.3. Meta-analysis of the clinical trials

Only 2 studies [11,12], involving 3822 patients with OHCA, were included in this analysis, with 3204 patients resuscitated with C-DACPR and 618 with V-DACPR. The mean age of patients treated with C-DACPR was 72.8 ± 11.5 years compared with 67.7 ± 13.8 years in the V-DACPR group (MD = 4.10; 95% CI: −2.07, 10.27; *I*^2^ = 98%, *p* = .19). The OHCA patient characteristics are presented in Table 2.

**Table 2.** Characteristics of patients with out-of-hospital cardiac arrest involved in the analysed studies.

Parameter	No. of studies	Events/participants		Events		Heterogeneity between trials		*p*-value for differences across groups
		C-DACPR	V-DACPR	OR	95% CI	*p*-value	*I*^2^ statistic	
Male sex	2	2019/3204	419/618	0.81	0.67, 0.97	.96	0%	.02
Comorbidities								
Diabetes	2	830/3204	142/618	1.15	0.94, 1.41	.55	0%	.18
Hypertension	2	1213/3204	213/618	1.14	0.95, 1.37	.99	0%	.16
Heart disease	2	613/3204	119/618	0.99	0.79, 1.23	.53	0%	.91
Cerebrovascular disease	2	336/3204	58/618	1.12	0.83, 1.52	.31	5%	.45
Cancer	1	245/1722	54/387	1.02	0.74, 1.41	NA	NA	.89
ECG								
VF/pVT	2	581/3204	208/618	0.47	0.34, 0.65	.11	62%	<.001
PEA	2	736/3204	136/618	1.07	0.76, 1.52	.11	60%	.68
Asystole	2	1894/3204	274/618	1.73	1.45, 2.06	.58	0%	<.001

C-DACPR: conventional audio-instructed dispatcher-assisted bystander cardiopulmonary resuscitation; V-DACPR: video-instructed dispatcher-assisted bystander cardiopulmonary resuscitation; OR: odds ratio; CI: confidence interval; ECG: electrocardiogram; VF: ventricular fibrillation; pVT: pulseless ventricular tachycardia; PEA: pulseless electrical activity; NA: not applicable.

Pooled analysis showed that the rate of prehospital return of spontaneous circulation (ROSC) was 11.8% in the C-DACPR group compared with 24.3% in the D-DACPR group (OR = 0.46; 95% CI: 0.30, 0.69; *I*^2^ = 66%; *p* < .001; Figure 2).

The rate of survival to hospital discharge with C-DACPR and V-DACPR amounted to 10.7% and 22.3%, respectively (OR = 0.46; 95% CI: 0.30, 0.70; *I*^2^ = 69%; *p* < .001). In the case of survival to hospital discharge with a good neurological outcome, statistically significant differences were noted between C-DACPR and V-DACPR (6.3% vs. 16.0%) (OR = 0.39; 95% CI: 0.23, 0.67; *I*^2^ = 73%; *p* < .001).

### 3.4. Meta-analysis of the simulation trials

**Figure 3.**
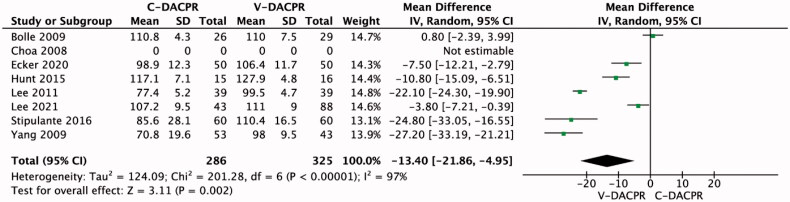
Forest plot for chest compression rate in the C-DACPR and V-DACPR groups. The centre of each square represents the weighted odds ratios for individual trials, and the corresponding horizontal line stands for 95% CI. The diamonds represent pooled results. C-DACPR: conventional audio-instructed dispatcher-assisted bystander cardiopulmonary resuscitation; V-DACPR: video-instructed dispatcher-assisted bystander cardiopulmonary resuscitation; SD: standard deviation; CI: confidence interval.

Seven studies reported chest compression rate. The mean chest compression rate per minute was 91.3 ± 22.6 for the C-DACPR group compared with 107.8 ± 12.6 for V-DACPR (MD = −13.40; 95% CI: −21.86, −4.95; *I*^2^ = 97%; *p* = .002; Figure 3). Adequate chest compression rate (Figures S3) [13,16,18,19] was observed in 29.2% of participants in the C-DACPR group and 55.0% in the V-DACPR group (OR = 0.30; 95% CI: 0.16, 0.57; *I*^2^ = 37%; *p* < .001; Table 3).

**Table 3.** Results of the simulation trials.

Parameter	No. of studies	Value		Events		Heterogeneity between trials		*p*-value for differences across groups
		C-DACPR	V-DACPR	OR	95% CI	*p*-value	*I*^2^ statistic	
Mean chest compression depth, mm	6	38.7 ± 14.3	41.8 ± 12.5	–2.67	–8.35, 3.01	<.001	98%	.36
Mean chest compression rate, /min	7	91.3 ± 22.6	107.8 ± 12.6	–13.40	–21.86, −4.95	<.001	97%	.002
Total no-flow time, s	3	126.2 ± 158.3	88.2 ± 117.7	3.28	–0.61, 7.17	.76	0%	.10
Adequate chest compression rate, *n*/total (%)	4	52/178 (29.2)	94/171 (55.0)	0.30	0.16, 0.57	.19	37%	<.001
Adequate chest compression depth, *n*/total (%)	4	39/178 (21.9)	53/171 (31.0)	0.69	0.32, 1.51	.16	42%	.36
Adequate hands position, *n*/total (%)	5	153/228 (67.1)	173/221 (78.3)	0.62	0.21, 1.81	<.001	79%	.39

C-DACPR: conventional audio-instructed dispatcher-assisted bystander cardiopulmonary resuscitation; V-DACPR: video-instructed dispatcher-assisted bystander cardiopulmonary resuscitation; OR: odds ratio; CI: confidence interval.

**Figure 4.**
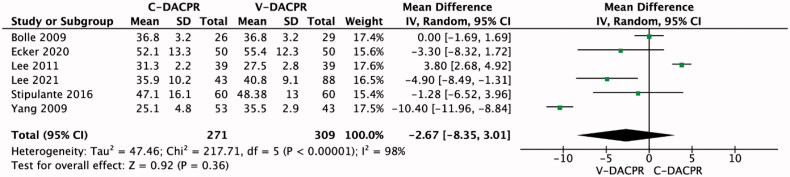
Forest plot for chest compression depth in the C-DACPR and V-DACPR groups. The centre of each square represents the weighted odds ratios for individual trials, and the corresponding horizontal line stands for 95% CI. The diamonds represent pooled results. C-DACPR: conventional audio-instructed dispatcher-assisted bystander cardiopulmonary resuscitation; V-DACPR: video-instructed dispatcher-assisted bystander cardiopulmonary resuscitation; SD: standard deviation; CI: confidence interval.

In the pooled analysis, chest compression depth in the C-DACPR and V-DACPR groups equalled 38.7 ± 14.3 and 41.8 ± 12.5 mm, respectively (MD = −2.67; 95% CI: −8.35, 3.01; *I*^2^ = 98%; *p* = .36; Figure 4). Four studies indicated adequate compression depth [13,16,18,19], which concerned 21.9% of patients in the C-DACPR group and 31.0% in the V-DACPR group (OR = 0.69; 95% CI: 0.32, 1.51; *I*^2^ = 42%; *p* = .36; Figure S4).

Adequate hands position was reported by 5 studies [10,13,16,18,19]. Pooled analysis showed that C-DACPR was related to adequate hands position in 67.1% compared with 78.3% for the V-DACPR group (OR = 0.62; 95% CI: 0.21, 1.81; *I*^2^ = 79%; *p* = .39; Figures S5).

## 4. Discussion

This is the first meta-analysis comparing C-DACPR with the more sophisticated V-DACPR. We have found that video instructions (V-DACPR) as compared with audio instructions only (C-DACPR) significantly improved all predefined and most relevant outcome parameters in the clinical trials enrolled, including the rate of out-of-hospital ROSC and survival to hospital discharge. Under simulated resuscitation conditions, V-DACPR compared with C-DACPR increased the adequate chest compression rate. This points at the high relevance of implementing V-DACPR to immediately improve OHCA outcomes in clinical settings.

Interestingly, in the clinical trials included in this meta-analysis, out-of-hospital ROSC and survival to hospital discharge rates were doubled with V-DACPR compared with C-DACPR. What was most impressive and of the highest clinical relevance, survival to hospital discharge with a good neurological outcome increased more than 2.5 times (6.3% vs. 16.0%) with V-DACPR. These results point to high applicability of V-DACPR in OHCA.

The meta-analysis of simulation trials further supports the positive effects of V-DACPR compared with C-DACPR, showing higher rates of adequate chest compressions. In some studies, V-DACPR was also more often associated with adequate hands position during CPR. All the CPR parameters investigated in the simulation trials are well known as quality indicators for good CPR and reperfusion and are related to better outcomes in clinical trials [27]. Furthermore, they may clearly explain the survival benefits detected in the clinical trials [11,12]. The use of V-DACPR improves the overall CPR quality.

Since OHCA is among the most common causes of death in industrialised nations, and early bystander CPR – as one of the BIG FIVE items in resuscitation [4] – is of crucial significance in markedly improving survival in the OHCA setting, our results may have a major impact on treatment strategies, as well as international recommendations for CPR and survival in cardiac arrest patients.

Lay bystanders are often overwhelmed in OHCA situations and may often not perform CPR or perform it inadequately [2]. Dispatcher-assisted CPR is associated with markedly improved outcomes [4]. Currently, C-DACPR has been already established in some areas and countries all around the world [2], and constitutes appropriate support for lay bystanders, resulting in apparent survival benefits [4]. The dispatcher, however, does not receive suitable feedback on whether their instructions lead to correct and high-quality CPR. Adequate feedback turns out much better with V-DACPR, which is not surprising. In several studies, the quality of CPR with V-DACPR was better in the context of compression rate and depth [10]. In addition, V-DACPR resulted in significantly fewer mistakes in choosing the correct hands position for chest compressions in some studies [10]. All this easily explains the impressive positive results revealed in our meta-analysis.

Despite the immense worldwide distribution of smartphone devices, and even though V-DACPR is not a new invention, there are still only a limited number of studies investigating its use for lay CPR [11,12]. To further support the findings of this meta-analysis, randomised controlled trials should be performed to compare the effects of V-DACPR and C-DACPR.

A potential limitation of our research may be the fact that until now, only 2 clinical studies, from the same group and country, have been published in the subject; both were retrospective analyses [11,12]. Nevertheless, the derived data and results from these clinical studies are intuitive and plausible, and largely comparable with the effects demonstrated in the simulation trials.

The data that served to perform the analyses presented in this paper are subject to various potential biases. In order to suggest a widespread adoption of the V-DACPR technology, changes in policies and regulations need to be further investigated with research based on a real-life comparison of V-DACPR and C-DACPR. These studies could be conducted with the use of software that may or may not allow applying V-DACPR when there are enough rescuers on the scene. Random selection of cases in which the dispatcher sees the image of the situation on the scene in comparison with the audio transmission alone would increase the quality of the research.

## 5. Conclusions

V-DACPR, as compared with C-DACPR, significantly improves CPR parameters in the clinical setting, including prehospital ROSC and survival to hospital discharge. Under simulated resuscitation conditions, V-DACPR increases the rate of adequate chest compressions as compared with C-DACPR.

## Data Availability

The data will be made available on request from the corresponding author.

## Abbreviations

OHCA: out-of-hospital cardiac arrest
CPR: cardiopulmonary resuscitation
C-DACPR: conventional audio-instructed dispatcher-assisted bystander cardiopulmonary resuscitation
V-DACPR: video-instructed dispatcher-assisted bystander cardiopulmonary resuscitation
OR: odds ratio
CI: confidence interval
MD: mean difference
ROSC: return of spontaneous circulation
